# Australian General Practitioners' Use of Diagnostic Lumbar Spine Imaging for Patients With Acute Low Back Pain: A Qualitative Study

**DOI:** 10.1002/msc.70099

**Published:** 2025-04-11

**Authors:** Tomas Rozbroj, Benjamin Reed, Denise A. O'Connor, Nicholas Gelber, Allison Bourne, Chris G. Maher, Rachelle Buchbinder

**Affiliations:** ^1^ School of Public Health and Preventive Medicine Monash University Melbourne Australia; ^2^ Royal Darwin Hospital Tiwi Australia; ^3^ Cabrini Research Malvern Australia; ^4^ School of Public Health University of Sydney Sydney Australia

**Keywords:** Australia, diagnostic imaging, general practice, low back pain, overdiagnosis, radiolog* report*

## Abstract

**Background:**

General practitioners (GPs) use of imaging for acute low back pain (LBP) is above guideline recommendations, and the reasons for this remain under‐researched. We examined the perspectives, expectations and information needs of Australian GPs requesting lumbar spine diagnostic imaging for patients presenting with acute LBP.

**Methods:**

We completed semi‐structured interviews with 12 GPs practising in Victoria, Australia. Transcripts were thematically analysed, and themes compared according to whether or not GPs reported they regularly requested imaging for LBP.

**Results:**

We identified four themes. (1) Besides responding to ‘red flags’, GPs' experiences of uncovering unexpected but serious findings on imaging for LBP as well as perceived external pressures motivated their defensive imaging practices. (2) While most were reluctant to request imaging for LBP, once requested, GPs escalated through imaging modalities and focused on the diagnostic benefit of their findings. (3) GPs supported the inclusion of epidemiological data on imaging reports, but (4) largely opposed imaging reports being written in plain language, believing reports to be clinician‐to‐clinician communications that patients would misunderstand. All GPs were aware of the limited utility of imaging for diagnosing LBP, and themes were similar between GPs who regularly requested imaging and those who did not. Factors other than knowledge of imaging efficacy for LBP seemed to play an important role in imaging requests.

**Conclusions:**

Our study identified key drivers of imaging use for LBP in primary care. The findings underscore that interventions targeting GPs addressing the overuse of imaging for LBP should transcend knowledge deficit models.

## 1 Introduction

Low back pain (LBP) was experienced by 4.4 million Australians in 2020 (Ferreira et al. 2023). While a third of patients do not seek care, LBP is one of the second most common reasons for visits to general practitioners (GP) (Britt et al. 2016). It accounts for 3.1% (Australian Institute of Health and Welfare 2020) of the 169 million annual GP visits in Australia (Royal Australasian College of General Practitioners 2024).

Between a quarter (Haas et al. 2023) and two‐thirds (Carey et al. 2015) of Australians presenting with LBP to primary care receive diagnostic imaging. This is at odds with guideline recommendations for best practice. Clinical guidelines recommend against early imaging in the large majority of LBP primary care patients: people not suspected of having serious and/or specific causes (Almeida et al. 2018; National Guideline Centre 2016; Qaseem et al. 2017). For this majority, imaging is unlikely to discern a definitive diagnosis (Hall et al. 2021) or a clear treatment direction (Foster et al. 2018), and may cause harm (Lemmers et al. 2019). Harms include needless treatment (Foster et al. 2018), which risks complications, adverse health outcomes and psychosocial and financial burdens (Graves et al. 2018; Lemmers et al. 2019; Wheeler et al. 2018). Inappropriate imaging also degrades healthcare system sustainability (Docking et al. 2022), and complex imaging has a notable deleterious effect on the environment (McAlister et al. 2022).

Researchers have sought to understand and address the overuse of imaging for LBP in primary care. A 2020 evidence synthesis, which included predominantly international research but also one Australian study focusing on GPs (Lin et al. 2016), found that clinicians requested imaging to identify the source of LBP pathology and as a form of defensive medicine (Sharma et al. 2020). Imaging is also used to aid discussions with patients (Sharma et al. 2020). A recent meta‐synthesis found that clinicians have mixed attitudes to providing imaging reports to patients, although the authors highlighted a need for more evidence about this (Alarifi et al. 2024). Another synthesis found that clinician's LBP guideline adherence is undermined by their beliefs that their experience supersedes guidelines, concerns that resisting requesting imaging may damage their relationship with patients, time poverty and lack of access to other diagnostic pathways (Slade et al. 2016). Many lumbar imaging reports describe benign clinical features (Farmer et al. 2024), and some limited evidence suggests that modifying the presentation of imaging report findings may improve care (Farmer et al. 2022).

Surprisingly, little is known about Australian GPs' reasons for requesting diagnostic imaging for LBP and how they then use imaging reports to manage LBP. Most insights come from international research (Sharma et al. 2020; Slade et al. 2016) and older Australian studies (Lin et al. 2016; Rogers 2002). Given that clinician practices vary across countries and that LBP management (Foster et al. 2018) and guidelines (Almeida et al. 2018) continue to evolve, recent Australian evidence is needed. It will aid the development of strategies that respond to the needs, attitudes and clinical realities faced by GPs in promoting adherence to LBP management guidelines.

Our study examined the perspectives, expectations and information needs of Australian GPs requesting lumbar spine diagnostic imaging for patients presenting with acute LBP.

## 2 Methods

The study was approved by the Cabrini Human Research Ethics Committee (approval number: 06‐27‐03‐17). The Monash University Human Research Ethics Committee provided governance approval (approval number: 9454).

The study was reported according to the COREQ (COnsolidated criteria for REporting Qualitative research) Checklist (Tong et al. 2007), included in Supporting Information S1: Appendix 1.

### 2.1 Methodological Orientation

This study is informed by a qualitative description theory (Neergaard et al. 2009). It emphasises that participants' self‐reported data capture meaningful if subjective insights into their experiences (Neergaard et al. 2009). It does not seek to apply an explicit theory to interpret these, and relies on emergent coding to systematically describe participants' perspectives (Stemler 2015). The strength of this approach is in highlighting GP own narratives. However, the lack of an explicit theoretical backbone can be considered a weakness for thematic analysis, which some argue is inherently informed by theory (Braun and Clarke 2021a). However, we concluded that for the relatively descriptive analysis in this study, weakness was minimised, and it was appropriate to take a descriptive approach.

### 2.2 Instrument

The interview guide is included in Supporting Information S1: Appendix 2. The interview guide was developed among the research team, which included leading Australian experts in LBP (CGM, RB), clinicians (BR, RB) and imaging experts (NG) as well as researchers with expertise in qualitative research (DAOC).

We selected a semi‐structured interview design to ensure that key topics were examined consistently across interviews, while giving us the flexibility to probe interesting threads of discussion. Interview topics comprised: GPs reasons for requesting diagnostic imaging for LBP, information sought from imaging, expectations from imaging reports, attitudes towards presenting imaging reports in lay terms, and; attitudes towards inclusion in imaging reports of epidemiological data conveying the prevalence of imaging abnormalities in people without LBP. For the last topic, GPs were shown examples of how epidemiological data could be presented in imaging reports. These examples were drawn from the Lumbar Imaging Reporting of Epidemiology randomised controlled trial protocol (Jarvik et al. 2015). Because our study population was time poor and difficult to recruit, instead of a traditional ‘pilot’, we used a reflexive approach to adjust the interview guide concurrently with interviewing (described in analysis).

### 2.3 Sampling

GPs were eligible to participate if they practised in Victoria, Australia and managed people with LBP in their practices. Recruitment occurred during September 2019 via purposive (email invitations) and snowball (word of mouth) sampling. The sampling frame comprised 800 GPs: 560 (70%) practicing in metropolitan areas and 240 (30%) practicing in rural/regional areas, reflecting Australian GPs metropolitan/non‐metropolitan workforce distribution (Department of Health 2018). We initially targeted 12 interviews but planned additional interviews if new themes continued to be identified. This reflected systematic evidence showing that ‘saturation’ can be reached within 9–17 interviews with a homogeneous population, with many studies reporting 12 interviews (Hennink and Kaiser 2022). GPs were reimbursed A$75 for participation.

### 2.4 Data Collection and Analysis

One author (BR) conducted all semi‐structured interviews. BR is male, was an undergraduate student trained in interviewing technique for this study, did not disclose any personal biases or interests about the study topic to participants, and had no prior relationship with them.

Interviews were conducted either in person or via audio calls, depending on what GPs found convenient. Interviews were audio‐recorded, transcribed verbatim and de‐identified. No field notes were made. Video‐recording was not considered because it raises additional ethical risks and practical challenges (Lobe et al. 2022), and we judged that the additional data gained would not provide enough value to our research aims to justify this.

Data was analysed thematically (Braun and Clarke 2006), coding for concepts in the data. Codes were developed iteratively from [re]reading the data and creating codes for ideas we identified in the data.

Initial analysis was done in parallel with interviews. This gave the interviewer (BR) an opportunity to reflexively adjust the interview schedule (Green et al. 2007). Additionally, in parallel with interviews, the interviewer familiarised himself with the data, developed a preliminary code frame, and undertook initial coding. He monitored data for repetition and for explanatory power to address our research aims (but not for ‘saturation’ in the neopositivist sense, which is argued to be a problematic concept in thematic analysis (Braun and Clarke 2021b)). He iteratively revised codes to reflect the ideas he found in the data as more interviews were completed.

We identified no new ideas in the data by the ninth interview, but three more interviews were conducted to develop a richer dataset. The initial interviews generated data that addressed our study aims, and therefore, no adjustments to the interview schedule were made.

Following data collection, BR optimised the code frame, completed line‐by‐line coding on the whole dataset, organised codes into broader themes, and checked and adjusted themes for internal homogeneity and data coverage. A second author (TR) completed further interpretative analysis of latent content, as well as comparative analysis of themes between GPs who did and did not regularly request imaging. Coding of 20% of the data was checked by one author (DAOC) for code frame fit, accuracy and consistency. We did not elicit feedback from participants about our findings. All authors contributed to the interpretation and reporting of findings. Exemplary comments that were consistent with themes and representative of the data were chosen.

## 3 Results

### 3.1 Sample

The sample profile is displayed in Table 1. No participants withdrew from the study. Interviews lasted approximately 1 h. Most participants were male (92%), trained in Australia (58%), practiced in a metropolitan area (83%) and reported no special interest in LBP (58%). The mean participant age was 59 years, and on average, they spent 29 years practicing in Australia.

**TABLE 1:** Participant profile (*N* = 12).

ID	Age	Gender	# Years practicing in Aus	Practice location	Where medical training completed	Special interest in LBP	Describe regularly imaging for LBP^a^
GP01	71	Male	48	Metro	India	Yes	No
GP02	38	Female	6	Metro	Ukraine	No	No
GP03	41	Male	8	Rural	China	No	No
GP04	33	Male	8	Rural	Australia	Yes	No
GP05	64	Male	40	Metro	Australia	No	No
GP06	64	Male	37	Metro	Australia	Yes	No
GP07	47	Male	24	Metro	Australia	No	No
GP08	73	Male	40	Metro	India	Yes	Yes
GP09	30	Male	7	Metro	Australia	No	No
GP10	80	Male	50	Metro	Singapore	No	Yes
GP11	64	Male	39	Metro	Australia	No	Yes
GP12	65	Male	41	Metro	Australia	Yes	No

^a^ As judged by researchers based on GPs qualitative responses.

### 3.2 Thematic Analysis

Four main themes were constructed: (1) GPs reasons for requesting imaging to diagnose LBP; (2) imaging modalities used and information sought; (3) support for inclusion of epidemiological data on imaging reports; and (4) lack of support for imaging reports in lay language. Exemplary comments were chosen based on how well they seemed to characterise the theme. They were slightly edited for grammar (i.e., to remove ‘umm’, repeated words and [inaudible]).

#### 3.2.1 GPs Reasons for Requesting Imaging to Diagnose LBP

Most GPs said that they did not regularly request diagnostic imaging for LBP. When GPs did request imaging, they appeared to have three main motivations. First, GPs requested imaging when patients presented with specific symptoms or reported traumatic injuries. For example, patients may have described falls that could have caused fractures, or GPs may have identified ‘red flags’, especially among high‐risk groups (e.g., elderly patients).

If they have had any recent trauma, like a fall, and—with high risk of fractures, like they are old and osteoporotic, then I will request a CT‐scan, which will pick up fractures better.

(GP04)

[I] normally [wait] four to six weeks, six to eight weeks [to order imaging]… assuming there's no incontinence and classic red flags, I suppose, loss of power and particularly focal neurology which is the majority of cases.

(GP07)

Second, they requested imaging for LBP to rule out rare but serious conditions. Across most interviews, GPs described specific experiences where imaging identified unexpected and serious findings, such as tumours, infections, or fractures. These experiences seemed to have informed GPs risk assessments about LBP, and motivated some to use imaging to rule out serious and rare conditions in subsequent patients presenting with LBP. Other GPs did not seem to use imaging to look for serious, rare pathology. Yet, a number of these GPs still described in detail instances when imaging led to unexpected serious findings, alluding to the significance of these risks in managing patients with LBP.

The trauma‐ it didn't sound like it was anything significant, the guy's not on any long‐term steroids or any other known risk factor where the bones might be weaker than what you would expect… but the x‐ray came back with a thoracic fracture… That really thumped me, and I was like ‘oh no have I been doing it all wrong’… So that was a highly unusual case, I don't know‐ I mean the guy ended up, the 22 year old, otherwise well guy‐ ended up going to the hospital because I think he had some arm pain or something like that…. That really made me think you know we probably shouldn't be black and white about ordering imaging. [The experience] was quite unusual and enlightening.

(GP02)

I remember a man climbing a ladder, and it would have been a painful, for the first time in his late 60s and he ended up having secondaries from a – a prostatic cancer… That was picked up on a plain x‐ray of the back. So, I still think they're quite useful in that.

(GP12)

They still can have the metastatic cancer… in the last five years I saw two cases already. One is very young, only 25 years old, come in with some outside back pain… I said I'd wait for one week maybe, just if no change to [get an] x‐ray. He didn't do it; he came back in two weeks' time with severe pain… Then they did CT scan straight away. That time they found out he's already had lymphoma involved metastatic in the spine here.

[GP went on to describe another example like the above]

Many times they come back and go, ‘the spine was okay but [we found] some mass somewhere’.

GP03

Thirdly, some GPs described requesting imaging because they were pressured to. Most commonly, this pressure was perceived to come from patients who expected to have imaging done. GPs generally said that they would meet patient requests for imaging, but some described attempting to dissuade them first. A few GPs mentioned requesting imaging for LBP following the suggestion of another healthcare professional (e.g., physiotherapist, radiographer). A couple of GPs mentioned imaging to meet the requirements for workplace injury insurance claims.

Patient reassurance. There is a strong need for some people to have x‐rays and a lot of the requests that patients come in with are from allied health people, physios, chiropractors, osteopaths… So I have a go, if a patient is really asking me ‘I've been told I've got to have a scan’.

(GP06)

[I sometimes order imaging in] certain cases to avoid confrontation. Sometime yelling, you know, the WorkCover people. You know that there is nothing over there. And to be very honest, if I order under duress ‐ the word duress is a bit strong ‐ I have to order an MRI, most of my MRI are normal.

(GP01)

#### 3.2.2 Imaging Modalities Used and Information Sought

GPs had nuanced reasons for choosing between imaging modalities (x‐ray, CT or MRI) to assess LBP. They evaluated the relative usefulness of imaging modalities for assessing the suspected cause of LBP by factoring in the patient's history, symptoms (i.e., whether patients experienced numbness or muscular atrophy) and the anatomical details of interest. GPs also considered practical and personal factors. These included patient preferences, suggestions from other clinicians (e.g., physiotherapist), procedural rules governing when an imaging modality can be requested, imaging costs, affordability for patients and the extent to which patients would be reimbursed. GPs tended to escalate imaging modalities until a perceived cause of LBP was identified.

I would start with just simple x‐rays. If they have already been investigated with x‐rays, the next step is a CT‐scan of the back. Again, if they have had any recent trauma, like a fall with high risk of fractures… then I will request a CT‐scan, which will pick up fractures better. And if they have Cauda Equina symptoms or signs, then I will request an MRI urgently.

(GP04)

A CT is rebated but some of the x‐ray clinics, in fact most of them probably, don't bulk bill for that unless they're a pension or healthcare card holder. So sometimes I think the decision may come down to cost in a patient's mind even though they mightn't air that with me. More often than not they're happy enough if we order a plain x‐ray first.

(GP11)

In imaging request forms for LBP, GPs included clinical questions, described the patient's symptoms and history, and some GPs specified the image framing and angle. They expected imaging reports to provide answers to their clinical questions in relation to the clinical details provided in the request. They sought specific findings describing anatomy, alignment and radiological changes, which were often perceived as pathological.

There appeared to be tension between some GPs' reasons for initially avoiding imaging and their expectations from imaging reports once requested. Most GPs were reluctant to initially request imaging for LBP because they were aware that anatomical changes visible on imaging may be normal for the patient's age. However, once they requested imaging, they focused on gaining detailed anatomical information, such as about alignment and scoliosis, to uncover the apparent cause of LBP.

To decide the relevance of imaging findings, some GPs sought an opinion from the radiologist about the clinical questions posed on the imaging request. But many GPs were also critical of imaging reports that included excessive or irrelevant detail, information that would scare patients, treatment recommendations or suggestions for further testing. GPs seemed to want radiologists to provide ‘the right amount’ of information to help GPs decide on the relevance of findings without being overly assertive and without taking away the locus of control from GPs. GPs were selective about using preferred radiological practices or radiologists.

[I want to know] Is the anatomy normal? Is there any variation from the normality in so far as the position of the spine. In other words, is there scoliosis, kyphosis, any congenital abnormalities, any additional vertebrae? Is there fusion of any vertebrae, like L5 on S1 or something like that? Absent arches? Congenital abnormalities? … Is there any localised features of trauma, any fractures, any abnormal swellings which usually don't get picked up on [x‐ray]? Degenerative changes, disc bulges, osteophytes, narrowing of disc spaces, narrowing of the neurological canals, exit canals?

[And I want] their conclusion. Most times I just glance through all these descriptions, and I like to see their final opinion. Sometimes it's good if they [give] some suggestions to follow up, but sometimes I think they tend to overdo it because … they restrict the capacity of the GP to do what he wants to do. [I like having their recommendations] if it is done sensibly with subtlety.

(GP06)

It irritates me a bit that they report things [that] when the patient reads they think that's the problem. So, you've got to spend a bit of time undoing that and teaching the patient and a lot of these things that are observed have nothing to do with their problem. You know, like Scheuermann's Disease and endplate this, and – you know, there's a lot of stuff that I wish they didn't put on there… I realise they have to describe what they see, but I like normal study, you know, when they write about – they sometimes describe scoliosis, I mean everybody's got a curve in their back, do you really need to mention that unless it's pathological? No.

[In response to a follow‐up question asking whether radiologists should report on the relevance of findings.]

Well, they – they often do and they'll say there is foraminal stenosis at L4/5 which probably accounts for the patient's symptoms… I don't need to read that really. I've got the patient in front of me. The radiologist is looking at images and he has got my little history, but he hasn't really seen or examined the patient.

(GP01)

GPs believed they understood imaging reports well, particularly X‐ray and CT scans. However, a few GPs reported difficulties in understanding some terminology, particularly in MRI reports, or believed that some reports described findings ambiguously.

There are a couple [of radiologists] that I don't think issue very good reports, they're a bit ambiguous and they're not very helpful. You read it and go ‘what, I'm not sure what that means, is it that or isn't it that’.

(GP06)

#### 3.2.3 Support for Inclusion of Epidemiological Data on Imaging Reports

GPs were asked whether they would value the inclusion of epidemiological information in imaging reports, including about the prevalence of ‘abnormal’ findings among different age cohorts without back pain. GPs were mostly supportive. They believed it would help them, or less experienced GPs than themselves, interpret imaging results. Some also thought that it would assist in patient communications.

I spend a lot of time advising the patient that a lot of the things that are reported by the radiologist are in fact normal findings of increased incidence with age. So, if it's already spelt out on the report, it sort of adds a bit more credence to what I'm telling them.

(GP06)

A few GPs did not support the inclusion of epidemiological information in reports because they thought patients would misinterpret it.

Very bad idea. Because statistics are statistics. To the individual, statistics don't mean a thing. If you are a 1% and you are it, that's 100% for you, so you can't say, you know, 99.99 out of 1000 people doesn't have this disease. But the guy who has it, it's 100% to him.

(GP10)

#### 3.2.4 Lack of Support for Imaging Reports in Lay Language

GPs were asked whether they would support imaging reports written in plain language to make them more accessible for patients. Most GPs were unsupportive. They believed that reports should be communications between healthcare professionals, and that GPs should mediate this information to patients. They were concerned that patients would misunderstand reports. For example, understanding the content superficially and missing important nuance, misunderstanding implications, and making erroneous conclusions.

Should the patient understand? I don't think so, because it's a communication between two health professionals.

(GP09)

It might simplify matters to the point where they read it as ‘it's all okay’. But we know that sometimes reports can be perfectly normal but there can still be pathology, so it might put that barrier for the patient to seek further medical advice because they've read the report and it said ‘it's okay’.

(GP02)

GPs were relatively more open to alternatives, such as giving patients a separate report or including a conclusion in lay terms at the end of the reports. While GPs were reluctant to make imaging reports easier to understand for patients, they routinely gave patients copies.

### 3.3 Comparison by Whether GPs Regularly Requested Imaging for LBP

Three‐quarters of the interviewed GPs reported that they did not routinely request imaging for LBP. All participants, including those who regularly used imaging for LBP, appeared to know that imaging had limited utility as a diagnostic tool for LBP. There were no identifiable differences in themes when we compared them between GPs who regularly requested imaging for LBP relative to those who did not. Both groups stated similar reasons for imaging, provided similar details on imaging requests, and sought similar information from imaging reports.

## 4 Discussion

We examined Victorian general practitioners' (GPs) attitudes to lumbar diagnostic imaging for people with acute low back pain (LBP). Our discussion focuses on considering the implications of our findings for working with GPs to promote more appropriate imaging requesting for LBP.

### 4.1 Risk Perceptions and Competing Demands Undermine Adherence to LBP Imaging Guidelines

All GPs in our study, regardless of whether they regularly requested imaging for LBP, were aware that early imaging has limited utility for diagnosis. However, similar to findings in previous research (Sharma et al. 2020; Slade et al. 2016), other drivers of imaging were evident, such as concerns about serious but rare findings, patient pressure and perceived value for patient reassurance.

These ‘other’ reasons for requesting imaging should be addressed in educational materials for clinicians. Serious pathology is rare. Older research indicates it is found in less than 1% of patients presenting to general practice with acute LBP in Australia (Henschke et al. 2009), while newer studies support this finding internationally (Street et al. 2020). Using imaging to rule it out is considered defensive medicine (Hong et al. 2017) and a contributor to low value care (Verkerk et al. 2022). Imaging for LBP also has limited utility as a patient reassurance tool. Patients feel most reassured when they receive an explanation about the cause of their symptoms (Holt et al. 2015), which imaging for LBP is unlikely to provide. According to older research, ruling out underlying pathology is shown to provide short‐term reassurance, but becomes less effective over time, and patients may seek further testing to attempt to identify the cause of their LBP (Linton et al. 2008).

Educational materials addressing inappropriate requesting of imaging for LBP should transcend ‘knowledge deficit’ approaches. ‘Knowledge deficit’ approaches assume that ignorance causes the unwanted outcome (Simis et al. 2016). For example, lack of knowledge about limited utility for imaging for diagnosing LBP drives low value care (Hall et al. 2021). But our results point to other important factors. GPs are generally aware that defensive medicine is harmful (Ries et al. 2022), but their anxiety and skewed risk perceptions seem to motivate them to request imaging anyway. Risk assessments are skewed towards vivid memories of tangible threats (such as unexpectedly finding cancers on an x‐ray or dealing with angry patients) (Ferrer and Mendes 2018; Kahneman 2011) and away from abstract threats, such as potential harms from low value care, which can often only be identified on a population level (Bulliard and Chiolero 2015). Similarly, factors such as ‘patient reassurance’, external pressures to order imaging or the psychology of utilising imaging reports once ordered are not driven by ignorance of facts. Rather, complex, behaviourally informed interventions are needed to address GP overuse of imaging for LBP. Some have already been developed (Jenkins et al. 2022; O'Connor et al. 2022). Further materials, including those to address risk perceptions and the psychology of patient reassurance, may be worth developing.

### 4.2 Once Imaging Is Requested, Do GPs Perceptions of Their Value Shift?

GPs appeared reluctant to request early imaging for people with acute LBP due to awareness of its limited utility. But they seemed to treat the findings of requests they did make as important diagnostic information, including to identify the cause of LBP. Our data could not adequately account for these apparent shifts in mindsets. Our theory that could underlie these shifts is that once GPs commit to imaging, they seek to maximise its utility and/or they move on from thinking about broader issues regarding its usefulness. GPs may also have limited scope for meta‐cognition within short, problem‐oriented consultation times (Henderson et al. 2016). Research examining these potential theories may help explain why GPs sometimes misattribute imaging findings for causes of LBP despite being aware of imaging limitations.

### 4.3 Lack of Support for Plain Language Imaging Reports

GPs were unsupportive of imaging reports being written in plain language, which added to the limited international evidence showing dissonant attitudes among doctors to giving patients unmediated access to imaging reports (Alarifi et al. 2024). Most GPs in our study nevertheless said they gave patients copies of imaging reports if patients requested them. It was unclear why GPs believed that plain language reports would preclude them from mediating imaging results. A minority of GPs also reported difficulties understanding imaging reports for LBP, so making these more comprehensible may benefit GPs. Furthermore, patients report wanting to be given imaging reports directly (Cabarrus et al. 2015), many access them when possible (Miles et al. 2016), and they regularly self‐educate about LBP outside of consultations (Yamaguchi et al. 2020). Patients misread jargonistic imaging reports for LBP to conclude these are more serious than is actually indicated (Farmer et al. 2021). Therefore, the merits of jargonistic language in imaging reports are questionable.

We lack evidence about the effect of giving patients easier‐to‐understand imaging reports for LBP. It is unknown how it will affect patients' treatment decisions, [mis]comprehension and satisfaction. During analysis, we got the impression that GPs were reluctant to further lose autonomy over the patient's management, but this is a conjecture and requires rigorous investigation. Our study showed that GPs were more supportive of including plain language statements at the end of LBP imaging reports, which may be a good compromise if the evidence support their use.

### 4.4 Inclusion of Epidemiological Data Is Acceptable, but Is It Effective?

The inclusion of epidemiological data in lumbar imaging reports has been thought to potentially improve the clinical management of LBP (Medalian et al. 2019), but there was uncertainty about clinician acceptance (Alarifi et al. 2024). Our research showed that most GPs would welcome it to aid patient communication and place the findings in context. GPs use imaging reports to aid patient communication and ease patient anxiety (Slade et al. 2016), while patients find a small degree of reassurance in receiving such information (Medalian et al. 2019). Therefore, the inclusion of epidemiological data seems like an obvious strategy. Unfortunately, evidence emerged since we collected our data suggesting that providing contextual information alone has little benefit for patients, although the benefit for clinicians was not assessed (Farmer et al. 2022).

### 4.5 Limitations

Our study had sampling limitations, including in the sampling frame, potential selection bias and a non‐representative sample profile. The sampling frame only included GPs practising in the state of Victoria, with metropolitan‐based participants concentrated in the middle‐class South‐East of Melbourne. Yet, there is healthcare variation across Australia (Australian Commission on Safety and Quality in Health Care 2021), limiting the broader applicability of our findings. The opt‐in recruitment method and snowball sampling may have favoured participation from GPs who were more available or who may have had an interest in LBP (5/12 reported having one). The sample was also skewed towards more experienced GPs, who older literature suggests may have had different treatment preferences compared with less experienced GPs (Choudhry et al. 2005). Almost all (11/12) of our participants identified as males. Previous research has shown that women clinicians tend to manage musculoskeletal conditions better than men (Tsugawa et al. 2017). Our findings offer important insights despite these limitations, but further work with representative samples would give us greater confidence in their validity.

A lack of patient or public involvement in the design of the study was also a potential limitation. While our study focused on GP perspectives and was designed using literature about patient perspectives, patients have different attitudes to imaging for LBP compared to GPs (Traeger et al. 2020). Not involving them in study design increased the risk we missed aspects of GP practice that matter to LBP patients.

The research was also limited to GP self‐reports of their practices. It is unknown whether GPs recall their practices accurately. A body of literature indicates that while clinicians and patients report largely similar things in describing LBP consultations, there is a degree of mismatch in their narratives (Sharma et al. 2020). Our findings could be strengthened by additional studies, including eliciting patient perspectives, directly examining imaging requests for LBP and/or observing clinical practice.

## 5 Conclusion

Our findings suggest that GPs are broadly aware of the limitations of early lumbar spine imaging in patients with acute LBP. However, other factors, including risk perceptions, cognitive frameworks and perceived interpersonal and systemic factors, promote non‐recommended use. Interventions that transcend knowledge‐deficit approaches to promote GP adherence to LBP guidelines may be needed.

## Author Contributions

R.B. conceived the research idea. All authors contributed to the study design or refinement. Data were collected by B.R. Analysis was completed by B.R., T.R., D.A.O.C. and R.B. The manuscript was drafted by T.R. and refined by all authors.

## Ethics Statement

The study was approved by the Cabrini Human Research Ethics Committee (approval number: 06‐27‐03‐17). The Monash University Human Research Ethics Committee provided governance approval (approval number: 9454).

## Conflicts of Interest

The authors declare no conflicts of interest.

## Supporting information

Supporting Information S1

## Data Availability

We state that we do not make the data reported in this article available because the dataset is confidential qualitative interviews that would be difficult to anonymise.
