# Supplementary material for: Australian General Practitioners' Use of Diagnostic Lumbar Spine Imaging for Patients With Acute Low Back Pain: A Qualitative Study
Source: Musculoskeletal Care. 2025 Apr 11;23(2):e70099. doi: 10.1002/msc.70099 (PMC11989193; doi:10.1002/msc.70099)
Supplement: Supplementary file 1 — Supporting Information S1 [file MSC-23-e70099-s001.pdf]

COREQ (COnsolidated criteria for REporting Qualitative research) Checklist

A checklist of items that should be included in reports of qualitative research. You must report the page number in your manuscript where you consider each of the items listed in this checklist. If you have not included this information, either revise your manuscript accordingly before submitting or note N/A.

Topic	Item No.	Guide Questions/Description	Reported on Page No.
**Domain 1: Research team and reflexivity**			
Personal characteristics			
Interviewer/facilitator	1	Which author/s conducted the interview or focus group?	4
Credentials	2	What were the researcher's credentials? E.g. PhD, MD	4
Occupation	3	What was their occupation at the time of the study?	4
Gender	4	Was the researcher male or female?	4
Experience and training	5	What experience or training did the researcher have?	4
Relationship with participants			
Relationship established	6	Was a relationship established prior to study commencement?	4
Participant knowledge of the interviewer	7	What did the participants know about the researcher? e.g. personal goals, reasons for doing the research	4
Interviewer characteristics	8	What characteristics were reported about the inter viewer/facilitator? e.g. Bias, assumptions, reasons and interests in the research topic	4
**Domain 2: Study design**			
Theoretical framework			
Methodological orientation and Theory	9	What methodological orientation was stated to underpin the study? e.g. grounded theory, discourse analysis, ethnography, phenomenology, content analysis	3
Participant selection			
Sampling	10	How were participants selected? e.g. purposive, convenience, consecutive, snowball	4
Method of approach	11	How were participants approached? e.g. face-to-face, telephone, mail, email	4
Sample size	12	How many participants were in the study?	5
Non-participation	13	How many people refused to participate or dropped out? Reasons?	5
Setting			
Setting of data collection	14	Where was the data collected? e.g. home, clinic, workplace	4
Presence of non-participants	15	Was anyone else present besides the participants and researchers?	4
Description of sample	16	What are the important characteristics of the sample? e.g. demographic data, date	6
Data collection			
Interview guide	17	Were questions, prompts, guides provided by the authors? Was it pilot tested?	appendix; 3-4
Repeat interviews	18	Were repeat inter views carried out? If yes, how many?	3-4
Audio/visual recording	19	Did the research use audio or visual recording to collect the data?	4
Field notes	20	Were field notes made during and/or after the inter view or focus group?	4
Duration	21	What was the duration of the inter views or focus group?	4
Data saturation	22	Was data saturation discussed?	5
Transcripts returned	23	Were transcripts returned to participants for comment and/or	5

Topic	Item No.	Guide Questions/Description	Reported on Page No.
		correction?	
**Domain 3: analysis and findings**			
Data analysis			
Number of data coders	24	How many data coders coded the data?	5
Description of the coding tree	25	Did authors provide a description of the coding tree?	No
Derivation of themes	26	Were themes identified in advance or derived from the data?	4-5
Software	27	What software, if applicable, was used to manage the data?	not relevant
Participant checking	28	Did participants provide feedback on the findings?	4-5
Reporting			
Quotations presented	29	Were participant quotations presented to illustrate the themes/findings? Was each quotation identified? e.g. participant number	results
Data and findings consistent	30	Was there consistency between the data presented and the findings?	n/.a
Clarity of major themes	31	Were major themes clearly presented in the findings?	results
Clarity of minor themes	32	Is there a description of diverse cases or discussion of minor themes?	results

Developed from: Tong A, Sainsbury P, Craig J. Consolidated criteria for reporting qualitative research (COREQ): a 32-item checklist for interviews and focus groups. *International Journal for Quality in Health Care*. 2007. Volume 19, Number 6: pp. 349 – 357

Once you have completed this checklist, please save a copy and upload it as part of your submission. DO NOT include this checklist as part of the main manuscript document. It must be uploaded as a separate file.

Appendix 2: interview schedule

- Do you routinely request diagnostic imaging for patients with acute low back pain? What type of imaging (x-ray, CT, MRI) and for what purpose?
- What information are you seeking when you request diagnostic imaging (x-ray, CT, MRI) for a person presenting with acute low back pain? Do you include this in the request?
- Do you discuss management options (conservative, pharmacological, interventional) prior to requesting imaging?
- Do you discuss with the patient the purpose of imaging and how it may or may not impact your management?
- What do you think about current request forms and procedures for requesting diagnostic imaging? Is it sufficient?
- Could this be improved? How?
- What information do you think is important to include on the imaging request for these patients?
- What specific information do you think should be presented in the imaging report (x-ray, CT, MRI) for these patients?
- How well do you understand imaging reports for acute low back pain?
- What can you and can you not understand about what is written?
- Can you comment on whether the report should be written in such a way to make it understandable to the patient? Why/why not?
- Some doctors and researchers have suggested adding epidemiological data to describe the prevalence of imaging findings in asymptomatic populations into imaging reports for acute low back pain. What do you think of this idea? *Show example*

- Do you think that current reporting methods for images requested for acute low back pain are sufficient? Why/why not?
- Would you make any suggestions as to how these reports could be improved?
- If someone was to develop a template to standardise reporting of diagnostic imaging of the lumbar spine (x-ray, CT, MRI) when requested for acute low back pain, what do you think should be included?
- Opportunity for GP to comment or raise points outside of immediate questioning

Demographic questions

- Age
- Sex
- Years practising in Australia
- Location of main practice (urban; regional/rural)
- Solo or group practice
- Location where medical training undertaken (Australia; overseas)
- Country and state of specialisation
- Special interest in LBP?
- Does your practice have a policy on requesting diagnostic imaging?
